# Investigating the ligand agonism and antagonism at the D_2long_ receptor by dynamic mass redistribution

**DOI:** 10.1038/s41598-022-14311-w

**Published:** 2022-06-10

**Authors:** Lisa Forster, Steffen Pockes

**Affiliations:** Institute of Pharmacy, University of Regensburg, Universitätsstraße 31, 93053 Regensburg, Germany

**Keywords:** Optical techniques, Receptor pharmacology, Receptor pharmacology

## Abstract

The signalling of the D_2_ receptor (D_2_R), a G protein-coupled receptor (GPCR), is a complex process consisting of various components. For the screening of D_2_R ligands, methods quantifying distinct second messengers such as cAMP or the interaction of the receptor with β-arrestin, are commonly employed. In contrast, a label-free biosensor technology like dynamic mass redistribution (DMR), where it is mostly unknown how the individual signalling pathways contribute to the DMR signal, provides a holistic readout of the complex cellular response. In this study, we report the successful application of the DMR technology to CHO-K1 cells stably expressing the human dopamine D_2long_ receptor. In real-time kinetic experiments, studies of D_2_R reference compounds yielded results for agonists and antagonists that were consistent with those obtained by conventional methods and also allowed a discrimination between partial and full agonists. Furthermore, investigations on the signalling pathway in CHO-K1 hD_2long_R cells identified the Gα_i/o_ protein as the main proximal trigger of the observed DMR response. The present study has shown that the DMR technology is a valuable method for the characterisation of putative new ligands and, due to its label-free nature, suggests its use for deorphanisation studies of GPCRs.

## Introduction

The dopamine D_2long_ receptor, a member of the rhodopsin family of GPCRs^1^ and one of the natural targets of the endogenous neurotransmitter dopamine, exerts its functions primarily by activating various subtypes of Gα_i/o_ proteins^2,3^. In consequence of alternative splicing the D_2_ receptor exists in three variants of which the short (in 18% of population studied) and long (79%) isoforms are the predominant forms with all isoforms coupling mainly through Gα_i/o_ proteins^4,5^. The D_2long_R is a highly interesting target molecule for the treatment of various neurological diseases such as Parkinson's disease (PD), addiction or schizophrenia^6^. It has been shown that D_2long_R signalling is multifaceted and comprises the activation of a variety of pathways^7^. By activation of the Gα_i/o_ protein, the D_2long_R inhibits the adenylyl cyclase and thus prevents the formation of cyclic AMP, leading to a decrease in the phosphorylation of protein kinase A (PKA) substrates^7^. Moreover, the Gβγ subunit, which dissociates from the heterotrimeric G protein after GTP is bound to the Gα subunit^8^, mediates an increase in cytosolic calcium by activation of phospholipase C (PLC)^9^. However, as has been shown in a neuronal cell line, an inhibitory effect on voltage gated calcium channels can also be detected^9^. In addition, the D_2long_R signals through β-arrestin2, a protein, that on the one hand is involved in the desensitisation of the receptor and on the other hand triggers G protein independent signalling^6^.

Traditionally, in G protein-coupled receptor (GPCR) targeted drug discovery, new compounds are characterised with respect to their pharmacological properties in binding and functional cell-based assays to determine ligand-receptor affinities and to quantify distinct intracellular messengers, respectively^10,11^. A very proximate technique to measure GPCR mediated G protein activation upon agonist stimulation is, for example, the [^35^S]GTPγS^3^ assay, which is usually performed with cell membrane preparations^12^. By contrast, assays utilising whole cells focus on the quantification of further downstream occurring intracellular second messengers, such as cyclic AMP^13^, inositol-1,4,5-trisphosphate (IP_3_)^14^ or Ca^2+^ ^15^, which are regulated by Gα_s_-, Gα_i_- or Gα_q_-coupled receptors. To monitor G protein independent signalling, namely the recruitment of β-arrestin, different approaches were described^16,17^. Among others, imaging-based^17^ or split-luciferase complementation assays^18–20^ are available, both requiring a modification of the GPCR of interest and/or β-arrestin to make the receptor-β-arrestin interaction conveniently detectable. In principle, the second messenger and effector recruitment assays can be applied for the investigation of orphan GPCRs. However, this is more time consuming compared to so-called label-free technologies because for the latter the G protein coupling specificity of the receptor does not have to be known (see below).

**Figure 1:**
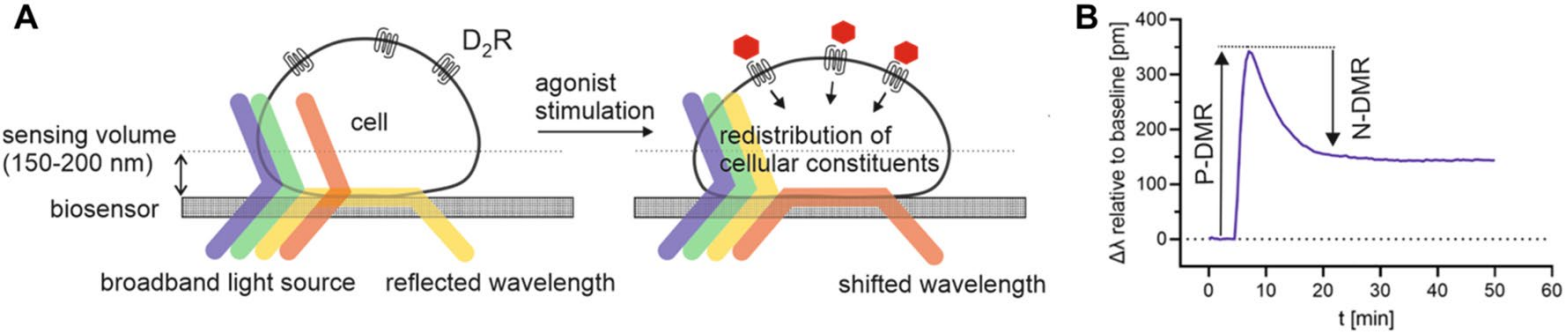
Schematic illustration of the DMR detection principle (adapted from Schröder et al.^31^). (**A**) Cells are grown in microplates that contain a resonant waveguide grating biosensor within the bottom of each well. The biosensor is illuminated by a broadband light source and the wavelength that is in resonance with the system is propagated and reflected. The resonance wavelength is a function of the refractive index near the surface of the biosensor. Stimulation of the cells can lead to a dynamic mass redistribution (DMR) of cellular constituents and subsequently a change in refractive index. This leads to a shift of the resonant wavelength in the pm range (Δλ), representing the readout of the DMR assay. (**B**) DMR that increases the optical density in proximity to the sensor surface results in a positive signal (P-DMR), whereas a decrease in optical density results in a negative signal (N-DMR), relative to the baseline^31^.

Concerning dopamine D_2_ receptor ligands, assays based on the quantification of cyclic AMP^21–23^, β-arrestin2 recruitment^19,24^ or [^35^S]GTPγS binding^25,26^ have been widely used for pharmacological characterisation. Having the complex signalling mechanisms of the dopamine D_2long_ receptor (or GPCRs in general) in mind, it appears to be advantageous to follow holistic approaches, i.e. label-free technologies, allowing the measurement of whole-cell responses to a ligand^11^. Dynamic mass redistribution (DMR) is a label-free technology that utilises an optical biosensor to measure the redistribution of cellular constituents upon receptor stimulation^27^. The biosensor used for measuring DMR is a resonant waveguide grating (RWG), consisting of a substrate and a cover layer with an embedded grating structure, and a layer of adherent cells that grow on the sensor surface^27^. As depicted in Fig. 1A, the bottom of the biosensor is illuminated by a broadband light source (825–840 nm) in a specific angle and most of the wavelengths are transmitted. The wavelength that is in resonance with the system is diffracted by the grating and couples into the grating layer, which acts as a waveguide. The light propagates within the layer until it is uncoupled again by diffraction. The wavelength that is in resonance with the system is, among others, determined by the refractive indices of the different layers, thus also by the local refractive index near the sensor surface^28^. Redistribution of cellular components, which has been reported as a complex endpoint of GPCR signalling^29,30^, results in changes in the refractive index next to the sensor surface^31^. This leads to a shift of the resonance wavelength which is recorded over time (cf. Fig. 1B)^32^. The electromagnetic field that is generated by the propagated light, the evanescent wave, shows a penetration depth in cells of about 150–200 nm, which is referred to as the sensing volume. Thus, only changes in mass distribution in the sensor-near portion of the cells are detected^33^.

Label-free techniques, such as DMR, are attractive because it is not necessary to know the G protein isoform coupling to the receptor of interest and a genetic engineering of the receptor is not required. This enables investigations under more physiological-like conditions, not least because there is no interference with cellular processes by the addition of chemical agents, often required for signal detection in conventional assays. On the other hand, the differentiation of individual signalling pathways plays an increasingly important role in current drug research as it is hoped that functional selectivity and the search for biased ligands will generate a reduced side effect profile^34^. Thus, ligands can appear in the different signalling pathways with different qualities of efficacies representing, for example, a G_i_-bias. This is also being discussed for D_2_-like receptors and is the subject of current research because an improved side effect profile is desirable especially in diseases such as PD and schizophrenia^35^. Just recently, detailed studies using histamine receptors and the human sweet taste receptor showed that G protein-dependent signalling pathways and G protein biased ligands can be studied with DMR^36,37^. Another strength of the DMR technique is its outstanding sensitivity, allowing the study of GPCRs at endogenous expression levels^30^. However, it should be kept in mind that extremely sensitive methods are particularly prone to error. Label-free readouts are often referred to as “black box” readouts, since the processes leading to the observed signal are not fully understood^38^. Therefore, specific antagonists or pathway inhibitors should always be included for the interpretation of data derived from DMR or other label-free assays.

In this study, well characterised dopamine D_2_R-like (partial) agonists and antagonists were investigated in a dynamic mass redistribution assay using CHO-K1 cells expressing the human dopamine D_2long_ receptor. The influence of different assay conditions on pharmacological parameters of the studied DR ligands and, furthermore, the contribution of different signalling components, such as G_s_, G_i/o_, G_q/11_ proteins or cytosolic Ca^2+^ were investigated. With our work, we aim to show that DMR is a valuable method for characterising receptors and ligands, which may be feasible to implement in a high-throughput format.

## Results and discussion

### Optimisation of assay conditions

Since the penetration depth of the biosensor is only about 200 nm, the adhesion of the cells to the sensor surface has a great impact on the sensitivity of the assay^39^. Considering that CHO cells have stronger adhesion to the microplates compared to HEK293T cells, they were selected for the realisation of this study. The expression of the receptor in the CHO-K1 hD_2long_R cell line was determined by radioligand saturation binding, shown in Supplementary Fig. S1 of the Supplementary Information (SI). Binding was saturable and dissociation constants were comparable with data obtained with HEK293T cells^20^. For the determination of pharmacological parameters under quasi physiological conditions in experiments involving intact cells, an assay temperature of 37 °C should be used. However, for different reasons (including minimal experimental influence on the DMR readout) explained in the Supplementary Information (Supplementary Figs. S2 and S3 and Supplementary Table S1), a lower assay temperature (28 °C) was considered useful. Therefore, all subsequent experiments were performed at 28 °C.

Another parameter investigated during the establishment of the assay was the cell density. The results of the experiments performed are discussed in the Supplementary Information (Supplementary Fig. S4 and Supplementary Table S2). For all subsequent experiments, cells were seeded at a density of 54,000 cells/well since this led to 80–90% confluency after about 24 h of incubation.

### Characterisation of reference ligands

After having determined the assay conditions for DMR measurements, concentration–response relationships of well-characterised reference DR agonists and antagonists were studied. The following agonists were investigated: the endogenous agonist dopamine, the full agonist quinpirole, pramipexole, a drug commonly employed in the treatment of Parkinson’s disease^40^, *R*-(−)-apomorphine, also a known Parkinson’s therapeutic^41^ described as partial agonist^42^, and aripiprazole, which is considered a prototype for third generation antipsychotics^43,44^. Quinpirole was used as the reference agonist defining the efficacies of the compounds as it exhibits higher chemical stability compared to dopamine^20^. A representative recording of the quinpirole induced change in wavelength shift from experiments performed with CHO-K1 hD_2long_R cells is shown in Supplementary Fig. S5 (SI). The stimulation of the cells with quinpirole elicited a positive concentration dependent DMR signal (Supplementary Fig. S5A, SI). Under the applied conditions, the observed change in wavelength shift increased rapidly, reaching a peak at about 3 min, followed by a rapid decline and stabilisation as a plateau above the baseline level. Comparable kinetic DMR profiles have been reported for other G_i/o_-coupled receptors expressed in CHO cells, such as the serotonin 5-HT_1B_ receptor^45^, the dopamine D_3_ receptor^46^ or the muscarinic M_2_ receptor^47^. As the DMR traces displayed clear maxima, the maximum change in wavelength shift (∆λ_max_; pm) was used to construct concentration–response curves (Supplementary Fig. S5B, SI). Data fitting according to a four-parameter logistic equation (cf. “Materials and methods”) afforded potencies (pEC_50_ values) and efficacies (E_max_) of the investigated D_2long_R agonists (Table 1).

**Table 1:** Functional data of D_2_R agonists (pEC_50_ and E_max_ values) and D_2_R antagonists (p*K*_b_ values) from DMR studies at CHO-K1 hD_2long_R cells. For comparison, functional data (β-arrestin2 recruitment) and binding data (competition binding) of D_2_R agonists and D_2_R antagonists are shown from Forster et al.^20^.

Compound	DMR					competition binding^a^		β-arrestin2 recruitment^a^		
	pEC_50_	E_max_ (%)	*N*	p*K*_b_	*N*	p*K*_iH_ or p*K*_i_	p*K*_iL_	pEC_50_	E_max_ (%)	p*K*_b_
Quinpirole	8.48 ± 0.05	100	9	–	–	7.90 ± 0.10^a^	6.11 ± 0.02^a^	7.55 ± 0.07^a^	100^a^	–
Dopamine	8.17 ± 0.10	110 ± 9	3	–	–	7.99 ± 0.16^a^	6.30 ± 0.07^a^	7.24 ± 0.04^a^	104 ± 3^a^	–
Pramipexole	8.71 ± 0.08	97 ± 0.1	3	–	–	7.59 ± 0.12^a^	6.00 ± 0.03^a^	8.19 ± 0.05^a^	86 ± 4^a^	–
Aripiprazole	6.44 ± 0.13	62 ± 10	3	–	–	8.32 ± 0.02^a^	–	6.65 ± 0.15^a^	8 ± 2^a^	–
*R*-(−)-apomorphine	9.25 ± 0.09	102 ± 5	3	–	–	7.48 ± 0.14^a^	–	7.77 ± 0.04^a^	87 ± 3^a^	–
( +)-butaclamol	–	–	–	n.d	–	9.14 ± 0.06^a^	–	–	–	8.29 ± 0.10^a^
Domperidone	–	–	–	9.16 ± 0.20	3	9.47 ± 0.07^a^	–	–	–	9.13 ± 0.09^a^
Haloperidol	–	–	–	9.17 ± 0.06	4	9.58 ± 0.13^a^	–	–	–	8.90 ± 0.05^a^
Nemonapride	–	–	–	9.24 ± 0.13	3	9.76 ± 0.08^a^	–	–	–	8.90 ± 0.05^a^
*S*-(−)-sulpiride	–	–	–	8.82 ± 0.23	3	7.51 ± 0.09^a^	–	–	–	8.86 ± 0.10^a^

Data represent means ± SEM from *N* or at least three independent experiments, each performed in triplicate. *K*_b_ values were derived by converting IC_50_ values to *K*_b_ values according to a modified Cheng-Prusoff equation, as described in “Material and methods”.

*n.d.*   not determined.

^a^Data described in Forster et al.^20^.

**Figure 2:**
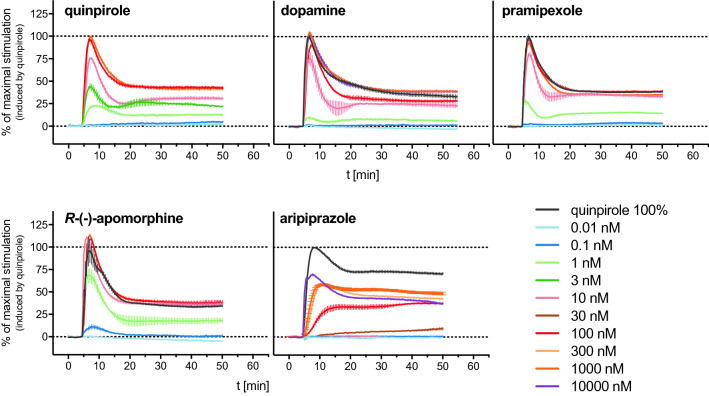
Representative DMR time courses of the concentration-dependent change in wavelength shift (Δλ; pm) induced by addition of the indicated reference agonists to CHO-K1 hD_2long_R cells. Data were normalised to the maximum wavelength shift induced by 1 μM quinpirole (100%) and a buffer control (0%). Shown are data (means ± SEM) from representative experiments out of three independent experiments, each performed in triplicate.

All agonists induced a positive DMR response in a concentration-dependent manner (Fig. 2), from which CRCs could be obtained (Fig. 4A). Quinpirole, dopamine and pramipexole appeared as full agonists in the DMR assay, yielding pEC_50_ values of 8.48, 8.17 and 8.71, respectively (Table 1). There are only little reports on the application of the DMR technique to D_2long_ receptors, but Brust et al.^48^ determined data for dopamine and pramipexole (EC_50_ values of 11 nM (dopamine) and 8.7 nM (pramipexole)) that were in very good agreement with the data obtained in the present study. *R*-(−)-Apomorphine, which was reported to be a partial D_2_R agonist in a [^35^S]GTPγS binding assay (E_max_ = 53%^42^ or 90%^49^ relative to dopamine, pEC_50_ = 7.66^42^ or 6.76^49^), appeared as full agonist in the DMR assay with a high potency of 0.6 nM (Table 1). Aripiprazole acted as a partial agonist in the DMR assay yielding an efficacy of 62% and a pEC_50_ value of 6.44, which was in good agreement with reported data (pEC_50_ = 6.23, obtained by conversion of the reported EC_50_ value, determined in a DMR assay)^48^.

**Figure 3:**
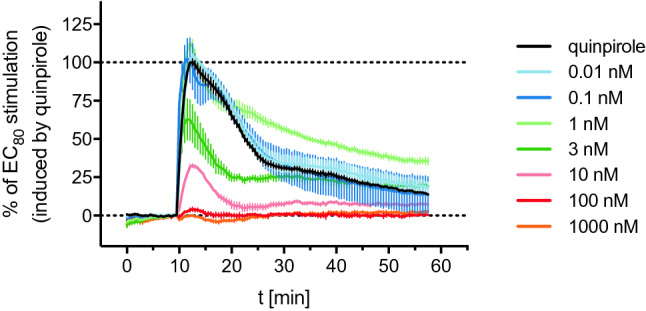
Exemplary DMR recording of haloperidol studied in antagonist mode. Before the addition of quinpirole at a concentration eliciting 80% of the maximal response (30 nM), the cells were incubated with varying concentrations of haloperidol for 30 min. Data were normalised to the maximum wavelength shift induced by 30 nM quinpirole (100%) and a buffer control (0%). Data represent means ± SEM from three independent experiments, each performed in triplicate.

**Figure 4:**
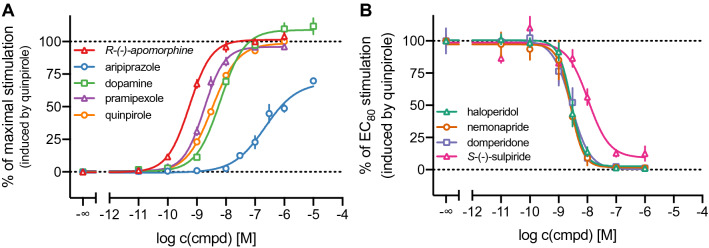
Characterisation of a set of reference DR agonists (**A**) and antagonists (**B**) in the DMR assay. CHO-K1 hD_2long_R cells were treated with varying concentrations of the indicated ligands and the DMR signal was recorded over a time-course of 60 min. The maximum wavelength shift (Δλ_max_) was used to construct concentration-effect curves. In agonist mode (**A**), the response was normalised to a solvent control (0%) and the maximum response induced by 1 μM quinpirole (100%). In antagonist mode (**B**), the cells were preincubated with various concentrations of antagonist for 30 min before quinpirole was added at a concentration (30 nM) that induces the response equal to 80% of the maximal response induced by 1 μM of quinpirole. The response was normalised to a solvent control (0%) and the response induced by 30 nM quinpirole (100%). Data represent means ± SEM of three independent experiments, performed in triplicate.

A selection of D_2_ receptor antagonists were studied for their ability to inhibit the quinpirole-induced DMR response mediated by the hD_2long_R, confirming the specificity of the response. The investigated antagonists were the antipsychotics haloperidol, nemonapride, *S*-(−)-sulpiride and the antiemetic drug domperidone. All four compounds, added 8 min after the addition of the agonist, antagonised the quinpirole induced DMR response in a concentration-dependent manner and comparable kinetic DMR traces were observed. Representative DMR time courses are shown in Supplementary Fig. S6 (SI). Higher concentrations of the antagonists led to a steep negative DMR response below the baseline level (ca. − 90%), which was reached after about 18 min. Subsequently, the DMR traces ascended slowly during the remaining recording. Lower concentrations of antagonists resulted in a less steep decline of the DMR traces. Similar DMR traces were also observed for the G_i_-coupled muscarinic M_2_ receptor, expressed in Flp-In CHO cells^31^. As demonstrated in Fig. 3, preincubation of the cells with the antagonists for 30 min prior to the addition of the agonist quinpirole resulted in time courses consisting entirely of positive signals. Hence, this kind of antagonist mode allowed the construction of concentration–response curves (Fig. 4B). The observed responses from experiments involving antagonists were less stable compared to the measurements with agonists and the wavelength shifts showed larger variations within individual triplicates (cf. Fig. 2 and Supplementary Fig. S6).

The inhibition curves obtained from the measurements with antagonists displayed Hill Slopes partially deviating strongly from unity (from −1.18 for *S*-(−)-sulpiride to −1.95 for nemonapride). Therefore, the typically used Cheng-Prusoff equation was considered inappropriate for the calculation of *K*_b_ values and a “more general” modified Cheng-Prusoff equation defined by Leff and Dougall^50^, that takes the Hill coefficient into account, was used to convert IC_50_ values to *K*_b_ values (as described in “Material and methods”). The obtained p*K*_b_ values are shown in Table 1. Unfortunately, no reference data of the studied dopamine D_2_ receptor antagonists obtained from DMR measurements were found in the literature for comparison.

All investigated D_2long_R ligands were tested for off-target activity in untransfected CHO-K1 cells. As shown in Supplementary Fig. S7 (SI) none of the agonists induced a DMR response in these cells. Regarding the antagonists, only nemonapride and haloperidol induced a slight negative DMR response. Structures of investigated ligands are given in Supplementary Fig. S8 (SI).

### Comparison of DMR data with results from conventional assays

**Figure 5:**
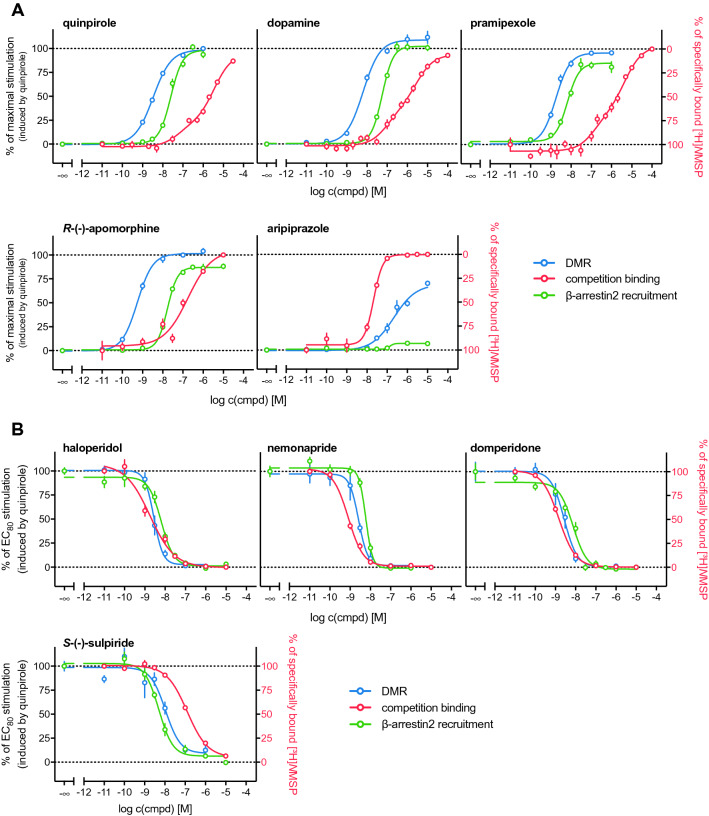
**(A)** Concentration–response and competition binding curves of selected dopamine D_2long_R agonists from different assay types. (**B**) Inhibition and competition binding curves of selected dopamine D_2long_R antagonists from different assay types. Data were normalised to the response induced by quinpirole at concentrations of 100 nM (β-arrestin2 recruitment) or 30 nM (DMR). The right Y-axis was inverted for illustration purposes. Competition binding experiments were performed on homogenates of HEK293T CRE Luc hD_2long_R cells. β-Arrestin2 recruitment assays were performed using whole HEK293T ELucN-βarr2 hD_2long_R-ELucC cells and the DMR measurements were carried out using whole CHO-K1 hD_2long_R cells. Data represent means ± SEM from at least three independent experiments, each performed in triplicate.

The results from the holistic DMR readout were compared with data obtained from radioligand binding and β-arrestin2 recruitment assays, which were described in Forster et al.^20^. In the case of the agonists quinpirole, dopamine and pramipexole binding constants for a high-affinity and a low-affinity binding state of the receptor were obtained in the radioligand competition binding assay. When comparing the p*K*_iH_ and p*K*_iL_ values with the potencies obtained in the different functional assays (DMR and β-arrestin2 recruitment) it appears that the pEC_50_ values correlate better with the *K*_i_-values for the high-affinity state (Table 1). This was in good agreement with the different reports on the high-affinity state of the D_2long_ receptor being the functionally relevant state^51,52^. The concentration–response curves (Fig. 5) show that the potencies determined in DMR measurements are higher compared to potencies obtained from β-arrestin2 recruitment. The intrinsic activities exhibited by these agonists were also highest for the DMR assay (Fig. 5A, Table 1). It has been reported that the sensitivity of DMR can be higher compared to traditional assays^47^ and DMR represents the most distal readout among the applied assays, therefore, the response can be highly amplified. However, it must be kept in mind that DMR measurements were performed with CHO cells, whereas competition binding and β-arrestin2 recruitment were determined using HEK293T cells. The rank order of potencies (pramipexole > quinpirole > dopamine) was the same in the “conventional” assays (focused on specific readouts) and the holistic technique.

*R*-(−)-Apomorphine, which was reported to act as D_2long_R partial agonist in a [^35^S]GTPγS binding assay^42^, exhibited efficacies of a full agonist in the DMR assay and appeared as a partial agonist in the β-arrestin2 recruitment assay (E_max_ = 87%) (Fig. 5A, Table 1). The pEC_50_ value of 7.77 determined in the β-arrestin2 recruitment assay is in the same range as the p*K*_i_ of 7.33 determined by radioligand competition binding. Measurement of the *R*-(−)-apomorphine-induced DMR yielded a significantly higher potency, a pEC_50_ value of 9.25. This could emerge from amplification of the response due to the distal readout, as already mentioned above. However, it should be questioned whether this is an adequate explanation for such a considerably higher potency determined by DMR measurements, especially with regard to lower discrepancies found for the other agonists. Since *R*-(−)-apomorphine did not elicit a DMR signal in untransfected CHO-K1 cells, as shown in Supplementary Fig. S7, it is unlikely that the observations result from stimulating other Gα_i/o_-coupled GPCRs that bind *R*-(−)-apomorphine with moderate to high affinity and are potentially expressed in CHO cells. Therefore, a plausible explanation for the observed high potency of *R*-(−)-apomorphine in the DMR assay could not be provided in the scope of the present study. Aripiprazole displayed a high affinity towards the D_2long_R, with a p*K*_i_ of 8.08, and pEC_50_ values ranged from 6.44 (DMR measurements) to 6.65 (β-arrestin2 recruitment assay). Its efficacies ranged from 8% in the β-arrestin2 recruitment to 62% in the DMR assay (Fig. 5, Table 1). Aripiprazole was reported to be a high affinity partial agonist at the D_2_ receptor^53^, which is in line with the obtained results.

The data of the antagonists analysed in the different assays are summarised in Table 1 and concentration-effect curves are shown in Fig. 5B. Generally, antagonistic activities determined in the β-arrestin2 recruitment and the DMR assay were in good agreement. As already observed for the potencies (pEC_50_) of the agonists, a tendency to slightly higher p*K*_b_ values in the label-free assay was observed for domperidone, haloperidol and nemonapride. For these compounds, and (+)-butaclamol, the affinities determined in radioligand displacement experiments are consistently higher compared to the p*K*_b_ values. *S*-(−)-Sulpiride represented an exception as its affinity (p*K*_i_) was markedly lower compared to the antagonistic activities (p*K*_b_) determined in the functional assays (Fig. 5B), which in turn were almost identical. In this study, p*K*_i_ values were determined in radioligand competition binding assays using a buffer that did not contain sodium ions, in contrast to the buffer used in DMR assays. It was previously reported that the absence of sodium ions negatively affects the binding affinity of substituted benzamide ligands like *S*-(−)-sulpiride to the dopamine D_2_ receptor^54^.

### Investigations on the signalling pathway in CHO-K1 hD_2long_R cells by DMR

#### Contributions of G_i/o_, G_s_ or G_q/11_ proteins to the D_2long_R mediated DMR response

**Figure 6:**
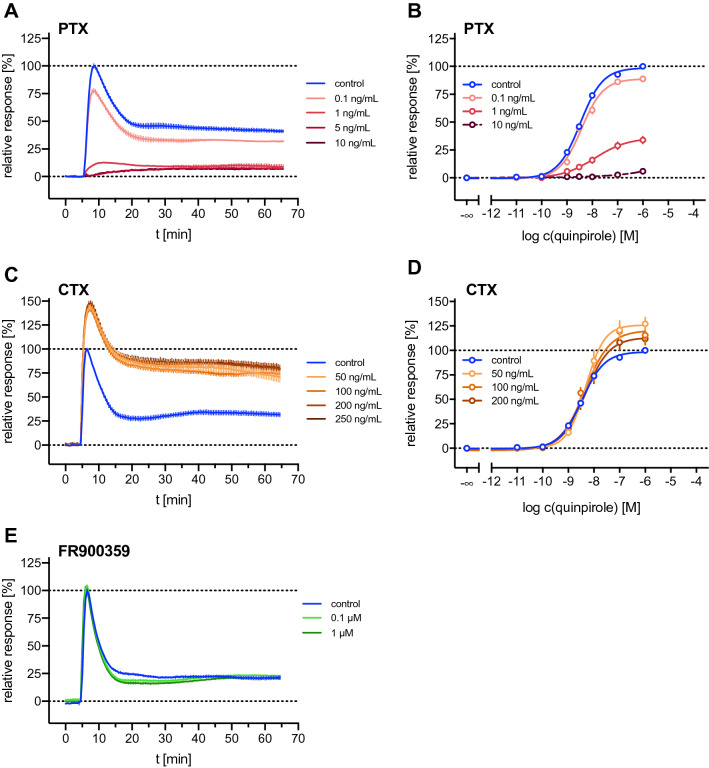
Effects of PTX, CTX and FR900359, capable of silencing G_i/o_, G_s_ and G_q_ signalling, respectively, on the quinpirole induced DMR response in CHO-K1 hD_2long_R cells. (**A**,**C**,**E**) Representative recordings of the quinpirole (1 μM) induced response of untreated cells (control) and cells pretreated with pertussis toxin (PTX) (**A**), cholera toxin (CTX) (**C**) or FR900359 (**E**). Data were normalised to the maximum change in wavelength shift induced by quinpirole (1 μM) observed in untreated CHO-K1 hD_2long_R cells (100%) and a buffer control (0%). (**B**) Concentration–response curves of quinpirole resulting from DMR measurements in the absence (control) or presence of PTX at different concentrations. Cells were pretreated with PTX for about 20 h. (**D**) Concentration–response curves of quinpirole resulting from DMR measurements in the absence (control) or presence of CTX at different concentrations. Cells were pretreated with CTX for about 20 h. In the case of blocking G_q_ signalling by FR900359 (**E**), cells were incubated with FR900359 for 2 h before the addition of quinpirole and subsequent measurement. Data in (**B,D)** represent means ± SEM of three independent experiments, each performed in triplicate.

Aiming at a deconvolution of the response pattern of CHO-K1 hD_2long_R cells observed in DMR measurements, distinct components of the signalling cascade were silenced using different pharmacological tools. For these studies, the cellular response was elicited by the D_2_R agonist quinpirole. The D_2long_R couples to G proteins of the G_i/o_ family^2^ and it was reported that DMR measures signalling effects downstream of G protein activation^47^. To investigate the contribution of G_i/o_ signalling to the DMR response, the Gα_i/o_ protein was blocked with pertussis toxin (PTX), which prevents the interaction of the respective receptor with the G protein by ADP-ribosylation of the Gα subunit^55^. Additionally, the effects of masking Gs and Gq signalling with cholera toxin (CTX) and the depsipeptide FR900359, respectively, were examined. CTX, like PTX, is an ADP-ribosylating toxin which inhibits the GTPase activity of Gα_s_ and thus transforms the Gα_s_ subunit into a permanently active state^56^. FR900359 suppresses Gα_q_ signal transduction by inhibiting the dissociation of GDP from the Gα subunit^57^. The effects of these compounds on quinpirole induced DMR responses in CHO-K1 hD_2long_R cells and the derived potencies and efficacies are shown in Fig. 6 and Supplementary Table 3. As expected, the application of PTX resulted in a concentration-dependent decrease in the DMR response (Fig. 6A), identifying G_i/o_ proteins as the main elicitor of the observed response. Increasing the PTX concentration from 5 to 10 ng/mL did not lead to a further suppression of the DMR signal, as can be seen in Fig. 6A. The remaining maximal effect of quinpirole observed in the presence of 10 ng/mL PTX was 6% (Supplementary Table 3), which was significantly different from zero (one-tailed t-test, *p* < 0.05). A slight rightward shift of the concentration–response curves of quinpirole appeared in the presence of increasing concentrations of PTX, resulting in decreasing pEC_50_ values (Fig. 6B, Supplementary Table 3). Masking G_s_ proteins with CTX led to an apparent increase in quinpirole efficacy without markedly shifting the concentration–response curves (Fig. 6C,D, Supplementary Table 3). As expected, the inhibition of the G_q_ protein with FR900359 did not show a pronounced effect on the quinpirole induced DMR response at any of the applied concentrations (Fig. 6E). These results supported the hypothesis that the observed DMR signal after stimulation of the cells with quinpirole is triggered by Gα proteins of the G_i/o_ family and that Gα_s_ and Gα_q_ subunits do not considerably contribute to the response. PTX, CTX or FR900359 on their own did not induce a DMR response in CHO- K1 hD_2long_R cells (Supplementary Fig. S9, SI).

#### Effect of elevated adenylyl cyclase activity on the D_2long_R-mediated DMR response

An increase in quinpirole efficacy observed after treating the hD_2long_R expressing CHO-K1 cells with CTX was also found when the cells were incubated with forskolin before the addition of the agonist quinpirole, as shown in Supplementary Fig. S10. Both agents, CTX and forskolin, lead to an increase in adenylyl cyclase activity, and thus to an elevated cellular cAMP level, but via distinct mechanisms. As mentioned above, CTX transfers an ADP-ribosyl residue to the Gα_s_ subunit, resulting in an inhibition of the GTPase activity of Gα_s_, which is thus constitutively active^56^. Consequently, cellular cAMP-levels are elevated^58^.

Forskolin increases the production of cAMP by directly activating the adenylyl cyclase^59^. It was reported that after prolonged forskolin treatment of D_2long_R expressing Ltk^−^ cells, quinpirole showed increased inhibitory efficacy in cAMP accumulation assays^60^. This effect was observed already after 1 h of forskolin treatment^60^. For the same Ltk^−^ cells, it was shown that forskolin treatment for 16 h caused an up-regulation of hD_2long_R expression, due to enhanced cAMP-dependent transcription^61^. Whether treatment of the CHO-K1 hD_2long_R cells with forskolin or CTX resulted in higher receptor expression was investigated by radioligand binding experiments. For this purpose, CHO-K1 hD_2long_R cells were treated with forskolin (1 μM) for 40 min or 20 h or with CTX (100 ng/mL) for 20 h and the binding of [^3^H]*N*- methylspiperone (1 nM) was compared to that of untreated CHO-K1 hD_2long_R cells. As shown in Supplementary Fig. S11 (SI), incubating the cells with forskolin for 40 min did not exhibit a marked effect on the receptor expression. However, prolonged treatment with forskolin for 20 h resulted in a strong increase in specific radioligand binding, being in line with the observations reported in the literature^61^.

Incubation of the cells with CTX for 20 h exhibited a less pronounced effect but the observed increase in specific [^3^H]*N*-methylspiperone binding was significant (*p* = 0.014, two-tailed t-test), indicating a slight up-regulation of the hD_2long_R. This could account for the increase in wavelength shift depicted in Fig. 6C. However, these results do not explain the marked increase in quinpirole-induced DMR response in CHO-K1 hD_2long_R cells observed after treating the cells with forskolin for 40 min and the underlying mechanisms remain unclear.

Forskolin alone mediated a negative DMR signal (Supplementary Fig. S10) with a minimum at about 5 min. The signal then increased and reached a plateau below the initial baseline, similar to the forskolin-induced DMR signal reported for CHO-K1 cells^62^.

#### Effects of calcium depletion on the hD_2long_R-mediated DMR response

**Figure 7:**
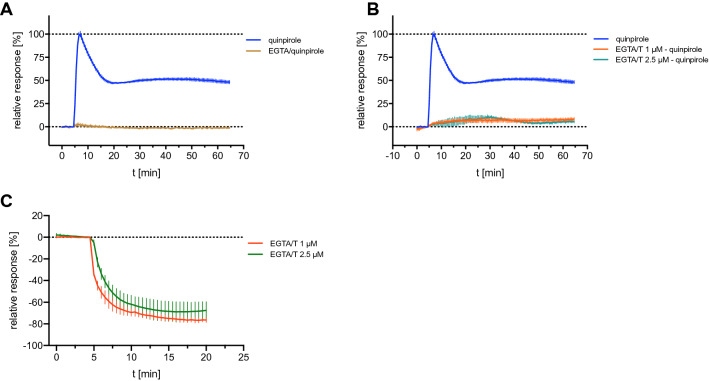
Effects of calcium depletion on the quinpirole-induced DMR traces of hD_2long_R expressing CHO-K1 cells. (**A**) Response induced by quinpirole (1 μM) in the absence or in the presence of EGTA (2 mM) in the assay buffer. (**B**) Cells were pre-incubated with thapsigargin (1 μM or 2 μM) for 20 min before the addition of quinpirole (1 μM). EGTA (2 mM) was present in the assay buffer. (**C**) Thapsigargin-induced effect on CHO-K1 hD_2long_R cells. The assay buffer was supplemented with EGTA (2 mM). In all experiments, after replacement of the medium by the EGTA-containing buffer, cells were allowed to condition in the pre-heated plate reader (28 °C) for 2 h. Data were normalised to the maximum change in wavelength shift induced by quinpirole (1 μM) observed in untreated CHO-K1 hD_2long_R cells (100%) and a buffer control (0%). Data shown are means ± SEM of representative recordings performed in triplicate of three independent experiments.

The data shown in Fig. 6E suggested that the Gα_q/11_ protein, mediating a strong increase in cytosolic calcium upon activation^63^, is not activated by the D_2long_R. However, it was reported that D_2long_R signalling increases intracellular calcium levels in a neuronal cell line through Gβγ-mediated activation of the phospholipase C resulting in the release of Ca^2+^ from intracellular stores^9^. Whether calcium also played a role in the formation of the quinpirole-induced DMR traces in CHO-K1 hD_2long_R cells was investigated by depleting the extra- or both the extra- and intracellular calcium pools. For this purpose EGTA was added solely or in combination with thapsigargin, a specific inhibitor of the endoplasmic reticulum Ca^2+^-ATPase^64^. EGTA is a metal ion chelating agent, which cannot permeate the cell membrane and shows higher specificity for Ca^2+^-ions compared to Mg^2+^-ions. Complexation of Ca^2+^ by EGTA leads to a Ca^2+^ depletion in the extracellular medium^65^. The combination with the membrane-permeable agent thapsigargin leads to an additional depletion of [Ca^2+^]_i_^66^. Surprisingly, both conditions completely abrogated the quinpirole-induced response of CHO-K1 hD_2long_R cells observed in DMR measurements (Fig. 7A,B). Figure 7C shows the signal induced by the addition of thapsigargin to the cells equilibrated in EGTA containing assay buffer. A rapidly decreasing negative DMR signal was observed, reaching a plateau after approximately 15 min. The lack of a detectable DMR response under conditions of calcium depletion was further investigated and the changes of [Ca^2+^]_i_ after stimulating the hD_2long_R expressed in CHO-K1 cells with quinpirole was explored by performing a Fura-2 calcium assay. Only high concentrations of quinpirole (1 μM and 10 μM) induced a low increase in intracellular Ca^2+^ concentration in CHO-K1 hD_2long_R cells over the buffer control (1.7-fold, cf. Supplementary Fig. S12, SI). For comparison, activation of the muscarinic M_3_ receptor, a G_q/11_-coupled receptor, results in an about tenfold increase in intracellular Ca^2+^-concentration in a Fura-2 assay using CHO-hM_3_R cells^67^. Consequently, the abrogation of the DMR response by treatment with EGTA and/or thapsigargin must have different underlying mechanisms. A potential effect of Ca^2+^ depletion on the binding of quinpirole to the receptor was considered a possible reason. Therefore, radioligand competition binding experiments were performed in the presence of EGTA (2 mM). However, the results showed that chelation of Ca^2+^ ions by EGTA had no marked effect on the binding of quinpirole to the hD_2long_R. (cf. Supplementary Fig. S13, SI).

In conclusion, extracellular or extra- and intracellular depletion of calcium resulted in conditions under which either the hD_2long_R cannot be activated or the hD_2long_R-mediated signalling cannot be detected by DMR measurements. Calcium is a ubiquitous intracellular second messenger^68^ which is involved in numerous cellular processes including the modulation of actin^69^. Since the DMR readout is based on actin-dependent cytoskeleton rearrangements or changes in cellular shape, depletion of Ca^2+^ could interfere with the measurement at this level. However, the underlying mechanism leading to a complete abrogation of the quinpirole-induced DMR response remains unclear.

## Summary and conclusions

The label-free DMR technology was successfully applied to CHO-K1 cells stably expressing the human dopamine D_2long_ receptor. Experiments performed at different temperatures showed that the kinetics of the agonist-induced DMR response was slightly slower at 28 °C compared to 37 °C and concentration–response curves (CRC) of D_2_R agonists were almost not affected by this temperature variation. Therefore, a temperature of 28 °C, being favorable when working with a device without automated liquid handling system, was applied for all subsequent investigations. A set of reference DR ligands was characterised using the DMR assay and robust CRCs were obtained for every studied (partial) agonist as well as high-quality inhibition curves for the antagonists. It could also be shown that the DMR technology allows a discrimination between partial agonists and full agonists. The signal induced by the agonist quinpirole could be antagonised by selective D_2_R antagonists, confirming that the observed DMR signal arose from a specific activation of the D_2long_R receptor. When comparing the agonistic and antagonistic potencies obtained from DMR measurements with the pharmacological parameters obtained from β-arrestin2 recruitment, the rank order was essentially the same. The pEC_50_ and p*K*_b_ values determined by the label-free technology tended to be higher than the values obtained from the β-arrestin2 recruitment assay, which may be explained by the high sensitivity and the distal readout of the DMR method. However, a different expression system was used for DMR measurements (CHO-K1 *vs*. HEK293T cells), which could also account for the observed differences. The utilisation of specific Gα_s_ Gα_i/o_ or Gα_q_ silencing agents identified the Gα_i/o_ protein as the main proximal trigger of the observed DMR response. However, the underlying mechanisms of the marked increase in wavelength shift after treatment of the cells with forskolin remained unclear. The present study showed that the DMR technology is a valuable method for the characterisation of receptors and putative new ligands complementary to canonical assays used to study ligand-receptor interactions. The label-free nature of the DMR techniques suggests its use for deorphanisation studies of GPCRs, provided that appropriate molecular tools such as specific pathway inhibitors, untransfected (wild type) cells and ideally also selective receptor ligands are included to verify the DMR signal specificity.

## Materials and methods

### Materials

Dulbecco’s modified Eagle’s medium/nutrient mixture F-12 Ham (DMEM/F-12) with phenol red, l-glutamine and sodium bicarbonate was purchased from Sigma (Taufkirchen, Germany). Fetal calf serum (FCS), trypsin/EDTA and geneticin (G418) were from Merck Biochrom (Darmstadt, Germany). Fura-2 AM was from Merck Biochrom (Darmstadt, Germany). Leibovitz’ L-15 medium (L-15) was from Fisher Scientific (Nidderau, Germany). Pertussis toxin was from Bio-Techne GmbH (Wiesbaden, Germany), FR900359 (UBO-QIC) was purchased from the University of Bonn (Germany) and cholera toxin was from Enzo Life Sciences GmbH (Lörrach, Germany). Thapsigargin was purchased from Tocris Bioscience (Bristol, United Kingdom), EGTA and forskolin were from Sigma-Aldrich GmbH (Taufkirchen, Germany).

### Cell culture

CHO-K1 hD_2long_R cells^70^ were a kind gift from Dr. Harald Hübner (Department of Chemistry and Pharmacy, Friedrich-Alexander-University, Erlangen). These cells were cultured in DMEM/F-12 supplemented with 10% FCS and 600 μg/mL G418 at 37 °C in a water-saturated atmosphere containing 5% CO_2_. Cells were routinely tested for mycoplasma contamination using the Venor GeM Mycoplasma Detection Kit (Minerva Biolabs, Germany).

### Dynamic mass redistribution assay

Dynamic mass redistribution monitoring was performed with an EnSpire multimode reader (Perkin Elmer, Waltham, USA), equipped with the Corning EPIC label-free technology using a resonance waveguide grating (RWG). CHO-K1 hD2_long_R cells were detached from a 25-cm^2^ flask by trypsinisation and centrifuged (22 ± 1 °C, 700*g*, 5 min). The pellet was resuspended in DMEM/F-12 containing 10% FCS and the cell density was adjusted to 0.6 × 10^6^ cells/mL. 90 µL of this cell suspension were seeded into 96-well EnSpire label-free sensor plates (cat # 6055408, Perkin Elmer), resulting in 54,000 cells per well. When 384-well plates (cat # 6057408, Perkin Elmer) were used, 50 μL of a cell suspension with a density of 0.32 × 10^6^ cells/mL were seeded, resulting in 16,000 cells per well. Cells were incubated at 37 °C in a humidified atmosphere containing 5% CO_2_ overnight. The next day, the culture medium was removed and the cells were gently rinsed with 70 μL (96-well plates) or 30 μL (384-well plates) of serum-free L-15 medium supplemented with 10 mM HEPES and 0.1% DMSO (assay buffer). Subsequently, 90 μL (agonist mode) or 80 μL (antagonist mode) of assay buffer were added per well in the 96-well plates. When 384-well plates were used, 45 μL of assay buffer were added. The cells were incubated in the assay buffer for 2 h in the pre-heated plate reader (28 °C or 37 °C for assay optimisation experiments) before a 5 min baseline was recorded. Afterwards, 10 μL (96-well plate) or 5 μL (384-well plate) of compound diluted in assay buffer (tenfold concentrated, 96-well plate) were added and DMR signals were acquired every 30 s for a period of 60 min. The readout is presented as the shift of resonance wavelength over time ∆λ(t), obtained by subtracting the last baseline measurement (λ(0)) from the raw data of the final read at time t (λ(t)): ∆λ(t) = λ(t) − λ(0). Concentration–response curves were constructed by plotting the maximum wavelength shift (∆λ_max_, pm) against the logarithmic ligand concentrations. The data were normalised to the maximum response induced by 1 μM quinpirole (100%) and the buffer control (0%) and fitted according to a four-parameter logistic equation (log(agonist) vs. response–variable slope, GraphPad Prism 9.0) to obtain EC_50_ values. Means were calculated from individual pEC_50_ values. Data obtained from experiments with antagonists were normalised to the maximum response induced by quinpirole corresponding to the EC_80_ (30 nM; 100%) and the buffer control (0%; L-15, supplemented with 0.1% DMSO) and fitted according to a four-parameter logistic equation (log(inhibitor) vs. response–variable slope, GraphPad Prism 9.0) to obtain IC_50_ values. IC_50_ values were used to calculate *K*_b_ values according to a modified Cheng-Prusoff equation, described by Leff and Dougall^35^:

$$K_{{\text{b}}} = {\text{ IC}}_{{{5}0}} / \, \left( {{2} + \left( {\left[ {{\text{QP}}} \right]/{\text{EC}}_{{{5}0}} } \right)^{{\text{n}}} } \right)^{{{1}/{\text{n}}}} - {1},$$

where [QP] corresponds to the applied concentration of quinpirole, [EC_50_] is the concentration of quinpirole producing 50% of the maximal response and n is the Hill coefficient of the concentration–response curve. Means were calculated from individual p*K*_b_ values.

## Supplementary Information

Supplementary Information.

## Data Availability

The datasets generated and analyzed during the current study are available from the corresponding authors on reasonable request.
