# Supplementary material for: Investigating the ligand agonism and antagonism at the D2long receptor by dynamic mass redistribution
Source: Sci Rep. 2022 Jun 10;12:9637. doi: 10.1038/s41598-022-14311-w (PMC9187652; doi:10.1038/s41598-022-14311-w)
Supplement: Supplementary file 1 — Supplementary Information. [file 41598_2022_14311_MOESM1_ESM.pdf]

Supplementary Information

Investigating the ligand agonism and antagonism at the D_{2long} receptor by dynamic mass redistribution

Lisa Forster[a,*], Steffen Pockes[a,*]

[a]Institute of Pharmacy, University of Regensburg, Universitätsstraße 31, 93053 Regensburg, Germany

Corresponding authors: lisa.forster@ur.de **(Lisa Forster),** steffen.pockes@ur.de **(Steffen Pockes)**

Contents

1 Optimisation of assay conditions S3

2 Characterisation of reference ligands S7

3 Investigations on the signaling pathway in CHO-K1 $D_{2long}R$ cells by DMR...... S10

4 References S13

1 Optimisation of assay conditions

Receptor expression

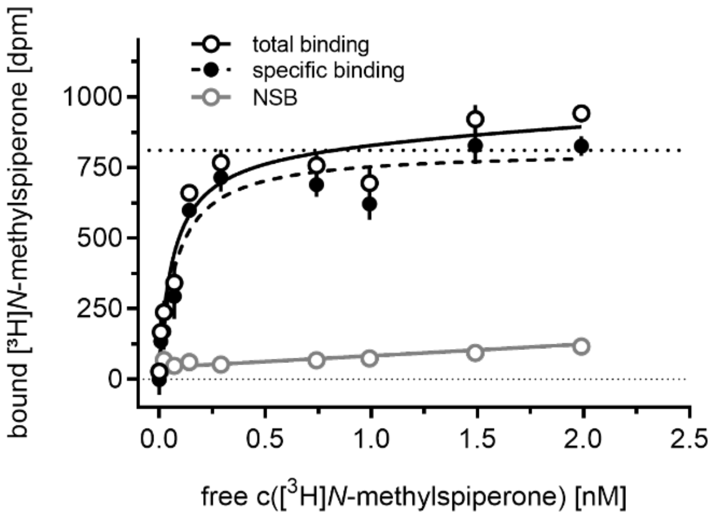

Supplementary Figure S1. Representative radioligand saturation binding curve obtained from saturation binding experiments with [^{3}H]*N*-methylspiperone at whole CHO-K1 hD_{2long}R cells. Experiments were performed as described by Forster et al.[1]. 35,000 cells per well were applied and non-specific binding was determined in the presence of a 2000-fold excess (+)-butaclamol. Shown is one representative experiment performed in triplicate of two independent experiments. A pK_d value of 10.20 ± 0.07 (mean ± SEM) was determined. Error bars of specific binding represent propagated errors. Error bars of total and non-specific binding represent the SEM.

Temperature

The biosensor used in the DMR technology is sensitive to the refractive index of the medium being in contact with the sensor surface[2]. Moreover, the refractive index depends on the temperature. A change of 1 °C results in a 24 pm shift of the reflected wavelength (according to the manufacturer), i.e. the temperature should be kept constant during the assay procedure. Before addition of the agonist, the microplate was kept in the plate reader for adaptation to the assay temperature and a baseline read was performed. Since the plate reader was not equipped with an automated liquid handling system, the receptor ligands were added manually outside of the device. To keep the temperature change minimal, measurements were performed at 28 °C and the resulting pEC_{50} values, the time point of the peak and the shape of the DMR traces were compared to those obtained from measurements at 37 °C.

Temperature has an impact on the fluidity of the cell membrane and it was reported that the mobility of membrane-anchored proteins increases with increasing temperature[3]. As shown in **Supplementary Figure S2** (SI), stimulation of the cells with an agonist results in a rapid shift in wavelength, which subsequently declines to almost the baseline level. In measurements performed at 37 °C (**Supplementary Figure S2**, **A** and **C**), the peak appears already after 0.5 - 2 min, whereas at 28 °C (**Supplementary Figure S2**, **B** and **D**), the kinetics is slightly slower and the maximum

wavelength shift is detected after 1.5 - 3.5 min. The decrease in temperature from 37 °C to 28 °C did not seem to have another impact on the shape of the DMR traces. To determine pEC_{50} values from the DMR recordings, data were converted to concentration-response curves (CRCs) shown in **Supplementary Figure S3**. By comparing the obtained pEC_{50} values (**Supplementary Table S1**), it becomes obvious that the potencies determined in experiments at 28 °C are lower than those obtained from experiments at 37 °C. To test for statistical significance, a t-test was performed and *p*-values < 0.05 were considered to indicate statistical significance. In the case of quinpirole, the pEC_{50} values obtained from measurements at different temperatures were significantly different (p = 0.03), whereas the pEC_{50} values determined for dopamine were not (p = 0.981). The results obtained for quinpirole indicate an impact of the assay temperature on the determined potencies. However, the rapid appearance of the peak may pose a problem when working without an automated liquid handling system (manual compound addition) as the addition of the compounds and starting the final read could take too long. Additionally it was demonstrated that membranes of live cells do not show phase transitions within a wide range of temperatures (14-37 °C)[3].

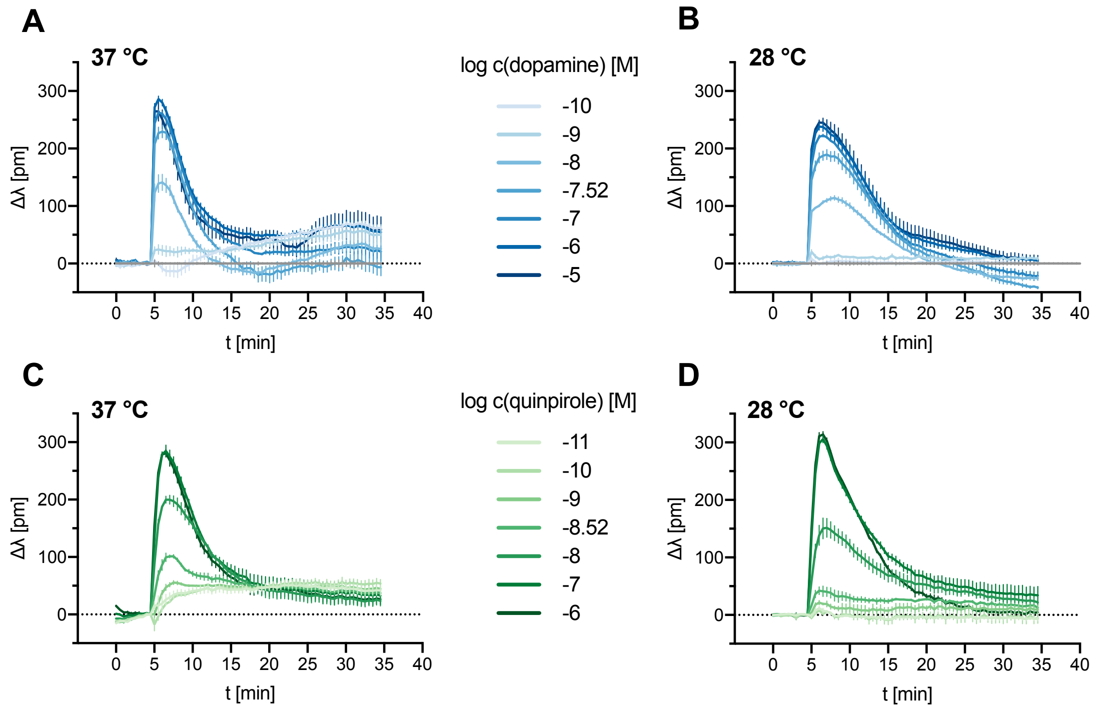

Supplementary Figure S2. Time courses of DMR experiments performed with CHO-K1 $D_{2long}R$ cells at 37 °C (**A**, **C**) or 28 °C (**B**, **D**). Cells were stimulated with the indicated concentrations of dopamine (**A**, **B**) or quinpirole (**C**, **D**) (added after 5 min baseline recording) and the wavelength shifts were monitored. Signals were corrected by subtraction of the vehicle control. Experiments were performed using 384-well microplates. Shown are means ± SEM of representative experiments, each performed in triplicate.

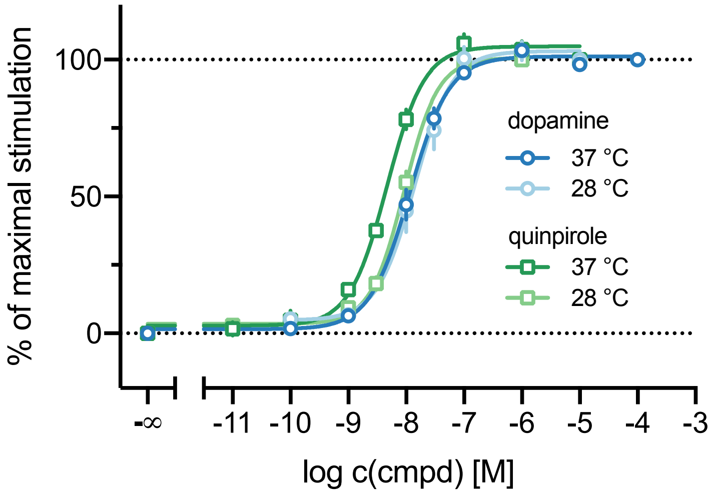

Supplementary Figure S3. Concentration-response curves resulting from DMR recordings at different temperatures. Relative maxima in wavelength shift ($\Delta\lambda_{max}$) are plotted against the logarithmic concentration of the respective agonist. Data were normalized to $\Delta\lambda_{max}$ induced by 100 µM dopamine or 10 µM quinpirole. Data are presented as means ± SEM from three independent experiments, each performed in triplicate.

Supplementary Table S1. pEC_{50} values determined by DMR measurements performed with CHO-K1 $hD_{2long}R$ cells at different temperatures.

**compound**	**pEC_{50} ± SEM**	
	37 °C	28 °C
dopamine	7.95 ± 0.18	7.87 ± 0.15
quinpirole	8.34 ± 0.04	8.03 ± 0.06

Data represent means ± SEM from three independent experiments, each performed in triplicate.

Cell Density

Cells were seeded at densities of 72,000, 54,000 or 36,000 cells/well into a 96-well plate and the DMR response was recorded upon stimulation with quinpirole (**Supplementary Figure S4**, **A**, SI). Concentration-response curves were constructed (**Supplementary Figure S4**, **B**, SI) and the resulting signal heights and pEC_{50} values were compared. As can be seen in **Supplementary Figure S4**, **A** (SI), the different cell densities did not have an impact on the maximal observed wavelength shift. The pEC_{50} values (**Supplementary Table S2**, SI) obtained from CRCs (**Supplementary Figure S4**, **B**, SI) decreased slightly with increasing cell density. For statistical analysis, the potencies were compared by a one-way ANOVA with Bonferroni's correction for multiple comparisons and revealed a significant difference only between values obtained from assays with 36,000 and 72,000 seeded cells/well ($p < 0.05$).

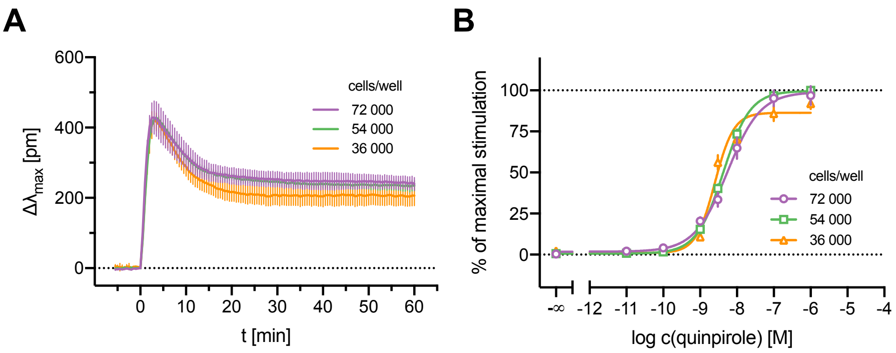

Supplementary Figure S4. Influence of the density of seeded CHO-K1 $D_{2long}R$ cells on the quinpirole induced response. (**A**) DMR recordings of CHO-K1 $D_{2long}R$ cells stimulated with 1 µM quinpirole. Data show one representative experiment performed in triplicate. (**B**) Concentration-response curves of quinpirole derived from DMR measurements at different cell densities. Data are presented as means ± SEM from three independent experiments, each performed in triplicate.

Supplementary Table S2. pEC_{50} values of quinpirole ($D_{2long}R$) determined by DMR measurements with varying cell densities.

seeded cells/well	pEC_{50} ± SEM (EC_{50}, nM)
36,000	8.58 ± 0.03 (6.0)
54,000	8.38 ± 0.04 (4.3)
72,000	8.25 ± 0.08 (2.7)

Data represent means ± SEM from three independent experiments, each performed in triplicate.

2 Characterisation of reference ligands

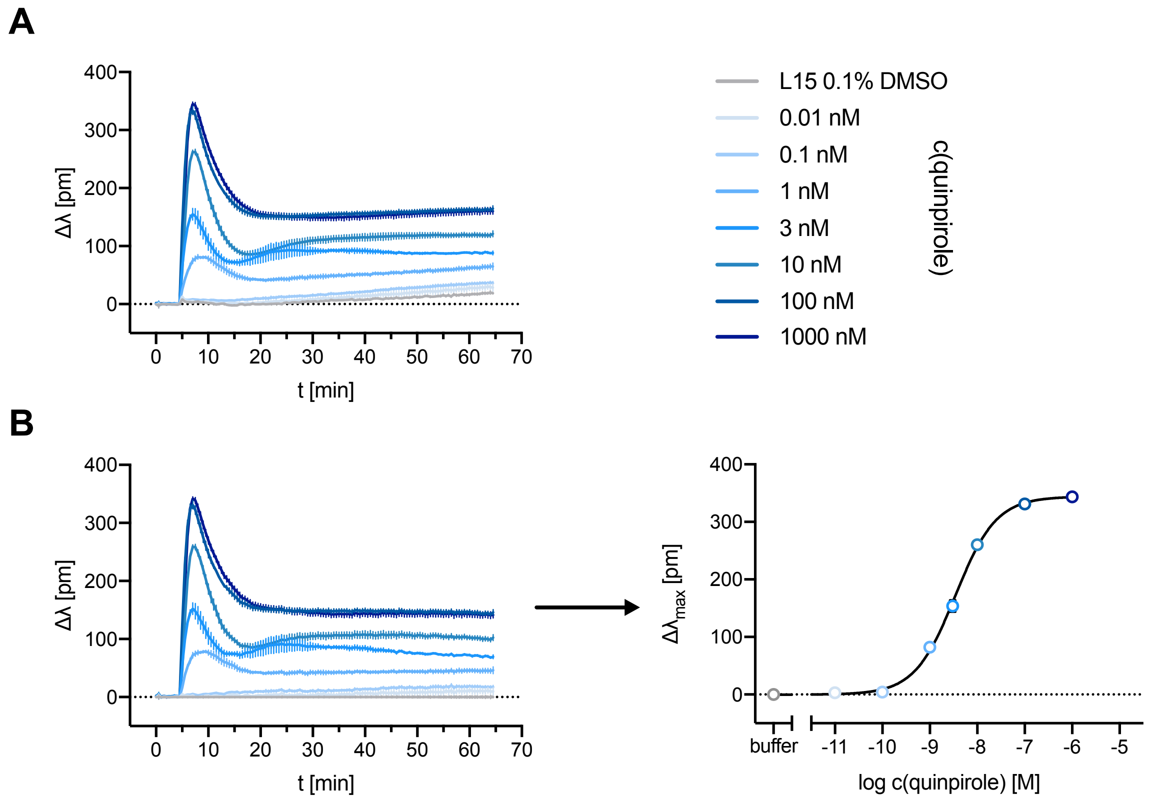

Supplementary Figure S5. Quinpirole induced responses of CHO-K1 $hD_{2long}R$ cells recorded by DMR and corresponding concentration-response curve. (**A**) Representative time courses of the change in wavelength shift after stimulating the CHO-K1 $hD_{2long}R$ cells with quinpirole at various concentrations (performed in triplicate). The measurement was performed at 28 °C in a 96-well microplate. (**B**) DMR traces from **A** corrected for the vehicle control and concentration-response curves generated by plotting the maximum change in wavelength shift ($\Delta\lambda_{max}$; pm) against the logarithmic concentration of quinpirole. Data are shown as mean ± SEM.

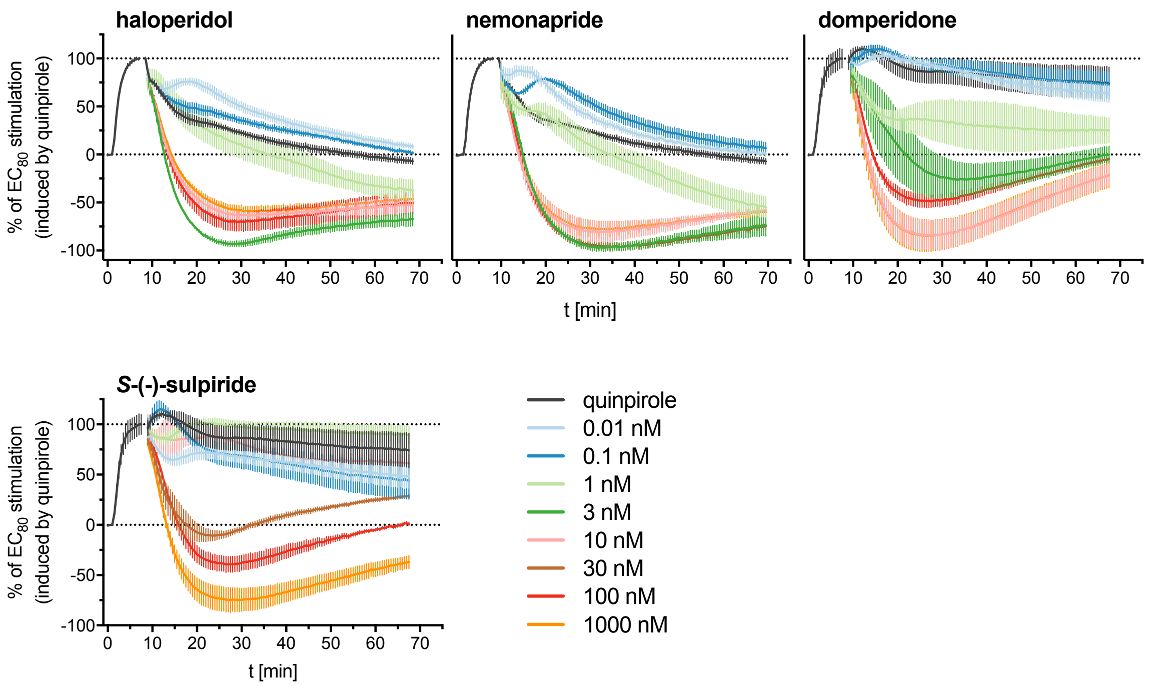

Supplementary Figure S6. Inhibition of the quinpirole-induced DMR response by selected dopamine receptor antagonists. CHO-K1 $hD_{2long}R$ cells were stimulated with quinpirole at a concentration eliciting 80% of the maximal response (30 nM) and the DMR signal was recorded for 8 min followed by the addition of varying concentrations of the indicated antagonist. Data were normalized to the maximum wavelength shift induced by 30 nM quinpirole (100%) and a buffer control (0%). Shown are means ± SEM of representative experiments performed in triplicate, out of at least three independent experiments.

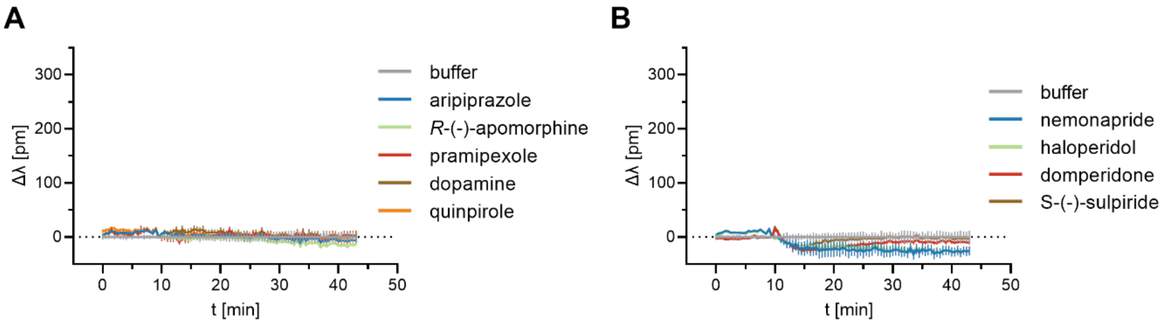

Supplementary Figure S7. Investigation of the effect of the indicated agonists (**A**) or antagonists (**B**) on CHO-K1 cells in the DMR assay. None of the agonists produced a shift in wavelength. Nemonapride and haloperidol showed a slight negative DMR signal (Δλ about -30 pm). Shown are representatives (means ± SEM) of three independent experiments, each performed in triplicate.

D_2R agonists

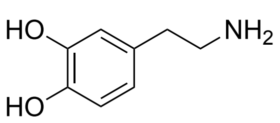

dopamine

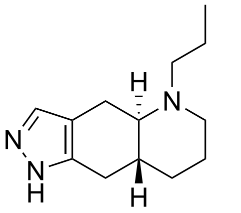

quinpirole

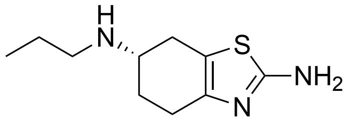

pramipexole

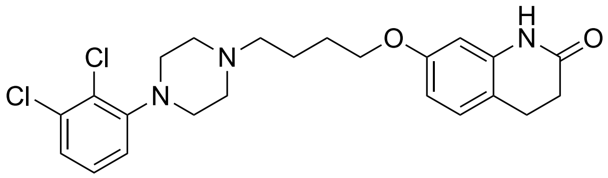

aripiprazole

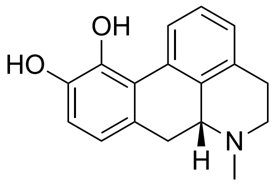

R-(-)-apomorphine

D_2R antagonists

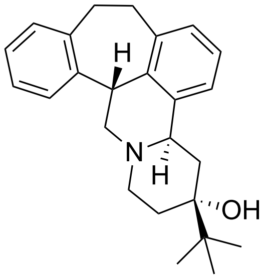

(+)-butaclamol

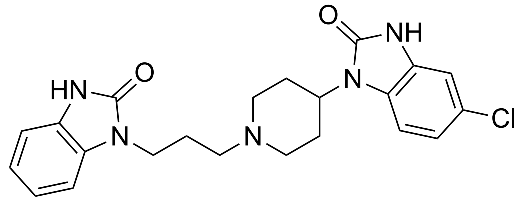

domperidone

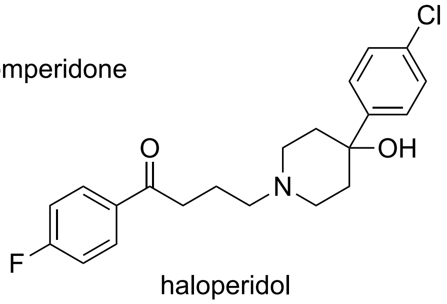

haloperidol

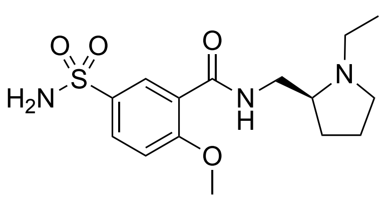

S-(-)-sulpiride

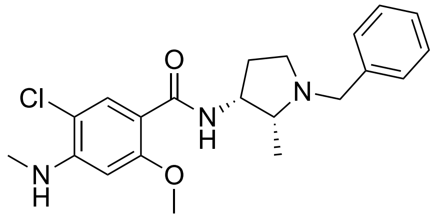

nemonapride

Supplementary Figure S8. Chemical structures of dopamine D_2 receptor agonists and antagonists applied in this study.

3 Investigations on the signaling pathway in CHO-K1 $D_{2long}R$ cells by DMR

Supplementary Table S3. Potencies of quinpirole determined at CHO-K1 $hD_{2long}R$ cells by DMR in the presence of pertussis toxin (PTX) or cholera toxin (CTX).

		**pEC_{50} ± SEM**	**% E_{max} ± SEM**
**PTX**	control	8.46 ± 0.05	100
	0.1 ng/mL	8.43 ± 0.05	89 ± 4
	1 ng/mL	7.88 ± 0.07	34 ± 5
	10 ng/mL	-	6 ± 1
**CTX**	control	8.46 ± 0.05	100
	50 ng/mL	8.36 ± 0.06	130 ± 10
	100 ng/mL	8.32 ± 0.13	130 ± 16
	200 ng/mL	8.34 ± 0.13	110 ± 9

Data represent means ± SEM from three independent experiments, each performed in triplicate.

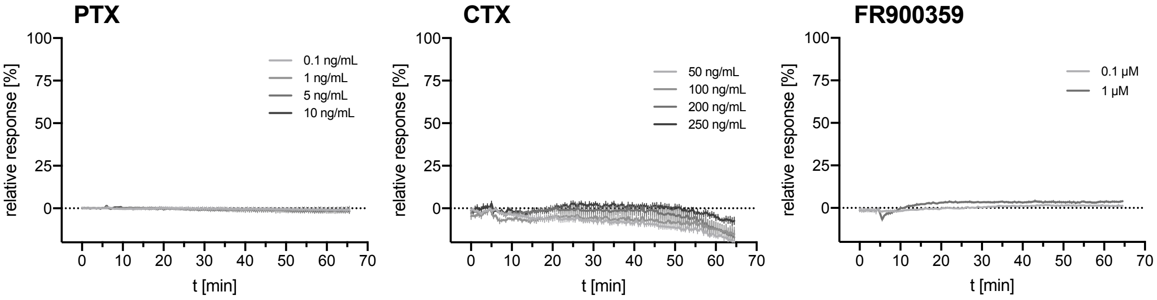

Supplementary Figure S9. Effect of PTX, CTX or FR900359 on the DMR response of CHO-K1 $hD_{2long}R$ cells. Shown are representatives (mean ± SEM) of three independent experiments, each performed in triplicate.

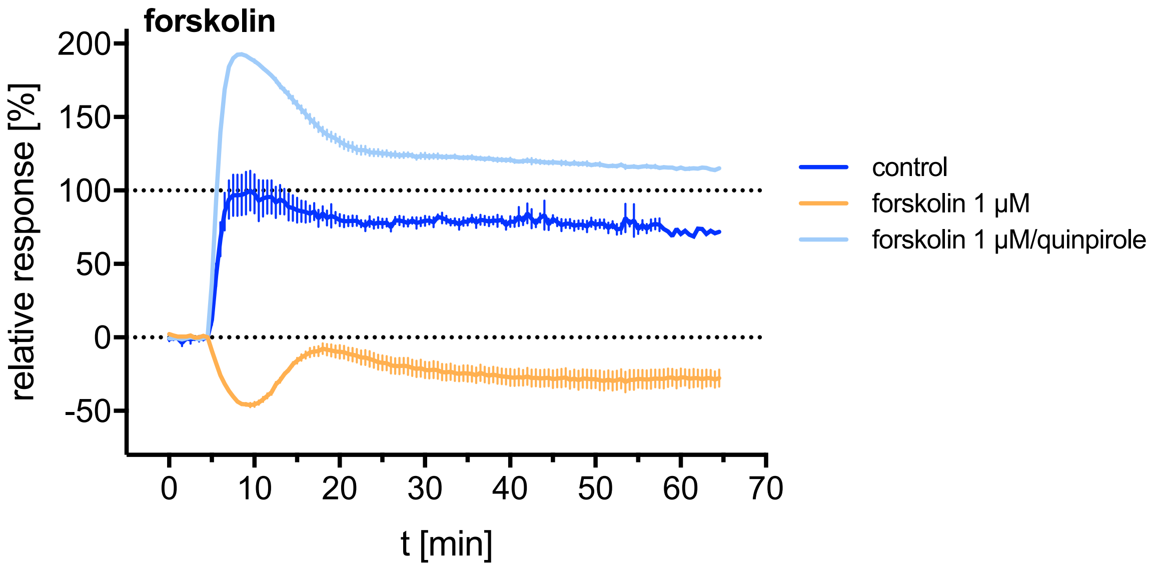

Supplementary Figure S10. Effect of forskolin on the quinpirole-induced DMR response of $D_{2long}R$ expressing CHO-K1 cells. The cells were stimulated with quinpirole (1 µM, control) or forskolin (1 µM) alone or incubated with forskolin (1 µM) for 40 min and then stimulated with quinpirole (1 µM). Data were normalized to the maximum change in wavelength shift induced by quinpirole (1 µM) observed in untreated CHO-K1 $hD_{2long}R$ cells (100%) and a buffer control (0%). Data shown are means ± SEM of representative recordings performed in triplicate of at least three independent experiments.

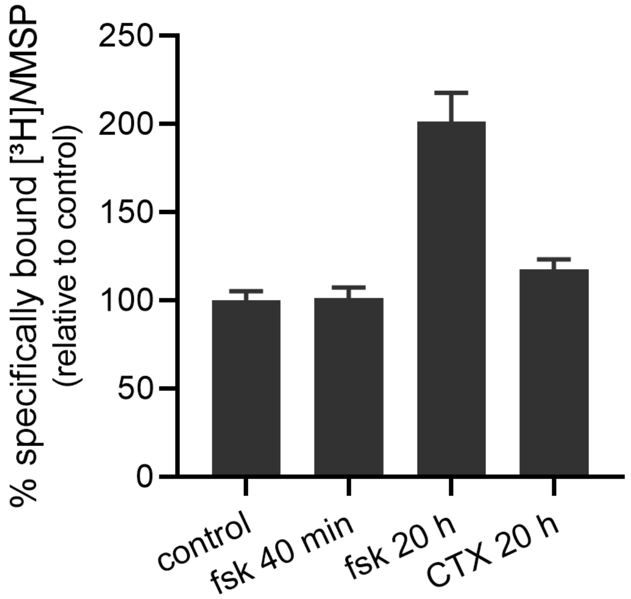

Supplementary Figure S11. Comparison of [^{3}H]*N*-methylspiperone binding to the $hD_{2long}R$ expressed in CHO-K1 cells after treatment with forskolin (fsk) or CTX with [^{3}H]*N*-methylspiperone binding to untreated cells. Cells were grown over night and treated with forskolin (1 µM) for 20 h or 40 min or with CTX (100 ng/mL) for 20 h. In a 96-well plate, 16 000 cells per well were incubated with [^{3}H]*N*-methylspiperone (1 nM) for 60 min. Non-specific binding was determined in the presence of (+)-butaclamol (2 µM). Data are normalized to radioligand binding to untreated cells (100%, control) and non-specific binding (0%). Presented are means ± SEM from two independent experiments.

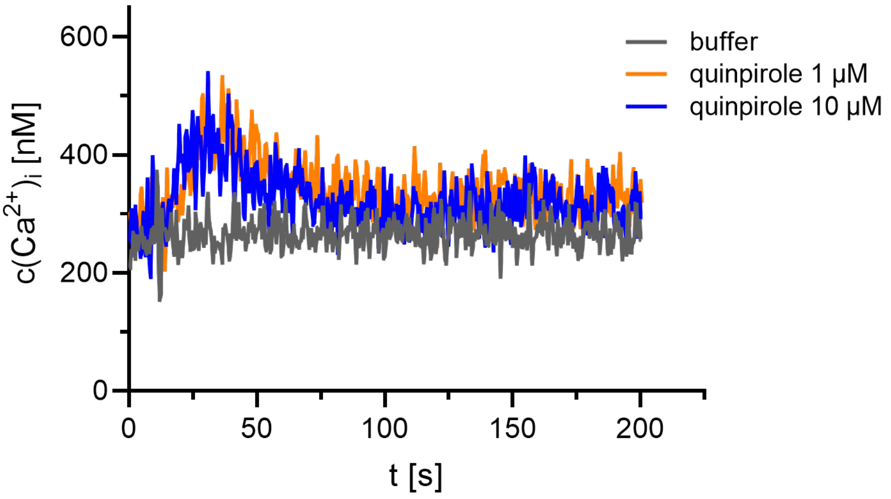

Supplementary Figure S12. Increase in intracellular Ca^{2+} upon stimulation of CHO-K1 $hD_{2long}R$ cells with quinpirole (10 and 1 µM) determined in a Fura-2 calcium assay. Shown is one representative experiment of three independent measurements. The Fura-2 calcium assay was performed as previously described with a LS50 B luminescence spectrophotometer (Perkin Elmer, Rodgau, Germany)[4].

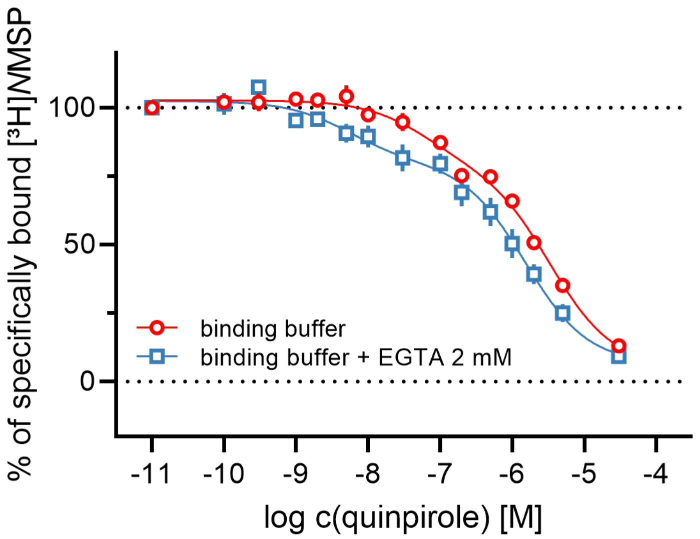

Supplementary Figure S13. Comparison of radioligand displacement curves obtained from competition binding experiments with $[^3H]$*N*-methylspiperone ($[^3H]$*N*MSP; 0.05 nM) and quinpirole under conditions as described by Forster et al.[1] (red line) and in the presence of EGTA (2 mM, blue line). The experiments were performed using homogenates prepared from HEK293T CRE Luc $hD_{2long}R$ cells[1]. Data are means ± SEM of three independent experiments, each performed in triplicate.

4 References

1. Forster, L., Grätz, L., Mönnich, D., Bernhardt, G. & Pockes, S. A Split Luciferase Complementation Assay for the Quantification of β-Arrestin2 Recruitment to Dopamine D2-Like Receptors. *Int. J. Mol. Sci.* **21**, 6103 (2020).
2. Fang, Y., Ferrie, A. M., Fontaine, N. H., Mauro, J. & Balakrishnan, J. Resonant waveguide grating biosensor for living cell sensing. *Biophysical journal* **91**, 1925–1940 (2006).
3. Lee, I.-H. *et al.* Live cell plasma membranes do not exhibit a miscibility phase transition over a wide range of temperatures. *The Journal of Physical Chemistry B* **119**, 4450–4459 (2015).
4. Müller, M. *et al.* Synthesis and Neuropeptide Y Y1 Receptor Antagonistic Activity of N, N-Disubstituted ω-Guanidino-and ω-Aminoalkanoic Acid Amides. *Archiv der Pharmazie* **330**, 333–342 (1997).
